# Proprotein convertase subtilisin/kexin type 9 expression is transiently up-regulated in the acute period of myocardial infarction in rat

**DOI:** 10.1186/1471-2261-14-192

**Published:** 2014-12-17

**Authors:** Yan Zhang, Jun Liu, Sha Li, Rui-Xia Xu, Jing Sun, Yue Tang, Jian-Jun Li

**Affiliations:** Division of Dyslipidemia, State Key Laboratory of Cardiovascular Disease, FuWai Hospital, National Center for Cardiovascular Diseases, Chinese Academy of Medical Sciences, Peking Union Medical College, BeiLiShi Road 167, Beijing, 100037 China

**Keywords:** Acute myocardial infarction, PCSK9, Rat

## Abstract

**Background:**

The proprotein convertase subtilisin/kexin type 9 (PCSK9) has been confirmed as a major factor regulating cholesterol homeostasis and has low-density lipoprotein receptor (LDLR) independent effects. In addition, the pathogenesis of acute myocardial infarction (AMI) involves lipids alteration and other acute phase responses. It remains unknown whether the PCSK9 expression is influenced by the impact of AMI. The present study aimed to investigate the changes of PCSK9 concentration using AMI rat model.

**Methods:**

AMI (n = 6-8 at each time point) or sham operated (n = 6) adult male rats model were used. Whole blood and liver tissue were collected at 1, 3, 6, 9, 12, 24, 48, and 96 hour (h) post infarction. The plasma PCSK9 concentration was measured by ELISA and lipid profiles were measured by enzymatic assay. The liver mRNA levels of PCSK9, LDLR, sterol response element binding protein-2 (SREBP-2) and hepatocyte nuclear factor 1α (HNF1α) were measured by quantitative real-time PCR.

**Results:**

The plasma PCSK9 concentration was increased from 12 h to 96 h (P < 0.05 vs. control). Paralleled with the enhanced plasma PCSK9 concentration, the hepatic PCSK9 mRNA expression was up-regulated by 2.2-fold at 12 h and 4.1-fold at 24 h. Hepatic mRNA levels of LDLR, SREBP-2 and HNF1α were all increased and lipid profiles underwent great changes at this acute period.

**Conclusions:**

We firstly demonstrated that PCSK9 was transiently up-regulated in the acute period of AMI, which is also driven by transcriptional factors, SREBP-2 and HNF1α, suggesting that the role of PCSK9 in myocardial injury may be needed further study.

## Background

Proprotein convertase subtilisin/kexin type 9 (PCSK9), originally discovered as a third gene involved in autosomal dominant hypercholesterolemia [1], has gained considerable attention over the past decade. A number of studies have demonstrated that, a high level of plasma PCSK9, caused by gain-of-function mutations in human or over-expression in animal model, exclusively resulted in increased serum concentration of low-density lipoprotein-cholesterol (LDL-C), and that the patients with gain-of-function mutations presented with increased cardiovascular risk [2–5]. Conversely, loss-of-function mutations in *PCSK9* and knockdown of *PCSK9* gene caused not only a low plasma level of PCSK9, but also a significant decreased level of LDL-C, accompanied by decreased cardiovascular risk [5, 6]. Recent studies have elucidated that PCSK9 is mainly synthesized by the liver and has been shown to bind to the low-density lipoprotein receptor (LDLR), subsequently promoting its degradation [7]. This process reduces the availability of LDLR, the major receptor mediating the clearance of low-density lipoprotein (LDL) particle, and results in increased plasma LDL-C levels. Since elevated LDL-C has long been established as a predominant risk factor for coronary artery disease (CAD), manipulating PCSK9 level would be a promising new treatment strategy [8].

The pathogenesis of acute myocardial infarction (AMI) is multifactorial, however, several studies have indicated that hyperlipidemia is a major risk factor accountable for 54% of population-attributable risk for AMI [9]. In addition, previous studies have reported that during the acute stage of AMI, serum lipids metabolism was severely affected [10]. Whereas PCSK9, an important factor regulating cholesterol homeostasis, has been reported to be associated with a variety of physiological and pathological factors dependent or independent of the LDLR, such as statins [11], fenofibrate [12], fasting [13], sex [14], periodontal infection [15], systemic inflammation [16], severe trauma injury [17] and the severity of coronary atherosclerosis [18]. However, it remains unknown whether the PCSK9 expression is influenced by the impact of AMI. Therefore, the aim of present study was to investigate the changes of both plasma and liver PCSK9 level using the AMI rat model.

## Methods

### Animal model

Adult male Sprague–Dawley rats (n = 6-8 at each time point), weighing 260-280 g, were acclimatized with a 12 hour (h) light/dark cycle at a controlled room temperature of 22–24°C, and rats were allowed free access to the regular and normal diet (fat content 4.62%) and clean drinking water for seven days before use. Myocardial infarction models were induced by ligation of the left anterior descending coronary artery (LAD) under ether anesthesia as previously described [19]. Electrocardiography was used to demonstrate ST elevation and thereby confirm the success of surgery. Sham operated animals (n = 6) underwent the same procedure except that no ligation was carried out. The experimental procedures were approved by the Institutional Animal Care and Use Committee of the FuWai Hospital and conformed to Guide for the Care and Use of Laboratory Animals from National Institutes of Health.

### Blood and tissue sampling

Prior to sacrifice, 2 mL fasting blood samples were collected from the tail vein at 1, 3, 6, 9, 12, 24, 48, and 96 h post infarction and transferred to K2 EDTA tubes. The blood samples were centrifuged, and the plasma was stored at -80°C until the analyses were performed. Immediately after blood sampling, the animals were sacrificed by an overdose of pentobarbital sodium. Tissue fragments from liver were collected and snap-frozen in liquid nitrogen and stored at -80°C.

### Blood sample measurements

Concentrations of serum total cholesterol (TC), triglycerides (TG), high-density lipoprotein-cholesterol (HDL-C), LDL-C, and free fatty acid (FFA) were determined on an automatic biochemistry analyzer (Hitachi 7150, Tokyo, Japan). Plasma PCSK9 concentration was measured using a high-sensitivity, quantitative sandwich enzyme immunoassay (Quantikine ELISA, R&D Systems Europe Ltd) according to our previous studies [20]. The lower limit of detection was 0.096 ng/ml. The white blood cell counts (WBCC) were determined using an automated blood cell counter (Beckman Coulter Ireland Inc Mervue, Galway, Ireland).

### RNA isolation, cDNA synthesis and quantitative real-time PCR

Following the manufacturer’s instructions, the total RNA of liver tissue was isolated using the Trizol reagent Kit (Invitrogen, USA) with the aid of an Omni Tissue Homogenizer, and then was measured by spectrophotometry at an absorbance of 260 nm, and designated the purity valid if the ratio of A260/A280 was in the range of 1.8 to 2.0. The integrity of the RNA was checked by denaturing agarose gel electrophoresis and ethidium bromide staining. First-strand cDNA was synthesized from 2 μg of total RNA using revert Aid First Strand cDNA synthesis kit (Promega, USA). Sequences of primers were given in Table 1. The abundances of key genes (PCSK9, LDLR, sterol regulator element binding protein-2 (SREBP-2), hepatocyte nuclear factor 1α (HNF1α)) and glyceraldehyde-3-phosphate dehydrogenase (GAPDH) mRNA were analyzed by real time quantitative PCR (RT-PCR). RT-PCR reaction was carried out with SYBR Premix ExTaq (TaKaRa Bio Inc. Japan) on 7500HT RT-PCR system (Applied Biosystems, Foster, CA, USA). Standard curves for each primer pair were generated by serial dilutions of cDNA from a reference sample and used for regression analyses. All PCR assays were performed in triplicate. The variance of the triplicate measurements was < 1%. Results were analyzed using the standard curve method by the sequence detection systems (SDS) software. All results were normalized against GAPDH.

**Table 1:** **Sequence of primers used in real-time RT-PCR**

Gene	Forward primer (5′ → 3′)	Reverse Primer (5′ → 3′)
PCSK9	CGGGAAGGACATCATCGG	GGTTCAGCATCATAGCCACAAT
LDLR	GATTGGCTATGAGTGCCTATGTC	GTGAAGAGCAGAAACCCTATGG
SREBP2	AGCATACCGCAAGGTGTTCC	CCAGGTGTCTACTTCTCCGTGT
HNF1α	ATGACACGGATGACGATGGG	ATGGGTCCTCCTGAAGAAGTGA
GAPDH	ACAGCAACAGGGTGGTGGAC	TTTGAGGGTGCAGCGAACTT

PCSK9 = proprotein convertases subtilisin kexin 9; LDLR = low-density lipoprotein receptor; SREBP-2 = sterol response element binding protein-2; HNF1α = hepatocytenuclear factor 1α; GAPDH = glyceraldehyde-3-phosphate dehydrogenase.

### Statistical analysis

Continuous variables were expressed as mean ± standard deviation (SD). One-way analysis of variance (ANOVA) followed by the Dunnett’s T3 test (unequal variances) or Bonferroni test (equal variances) were performed to test the differences among sham and AMI groups. A P value of less than 0.05 was considered statistical significance. The SPSS 19.0 statistical software package (SPSS Inc., Chicago, IL, USA) was used for all of the statistical analysis.

## Results

### Plasma concentration of PCSK9 and WBCC

**Figure 1:**
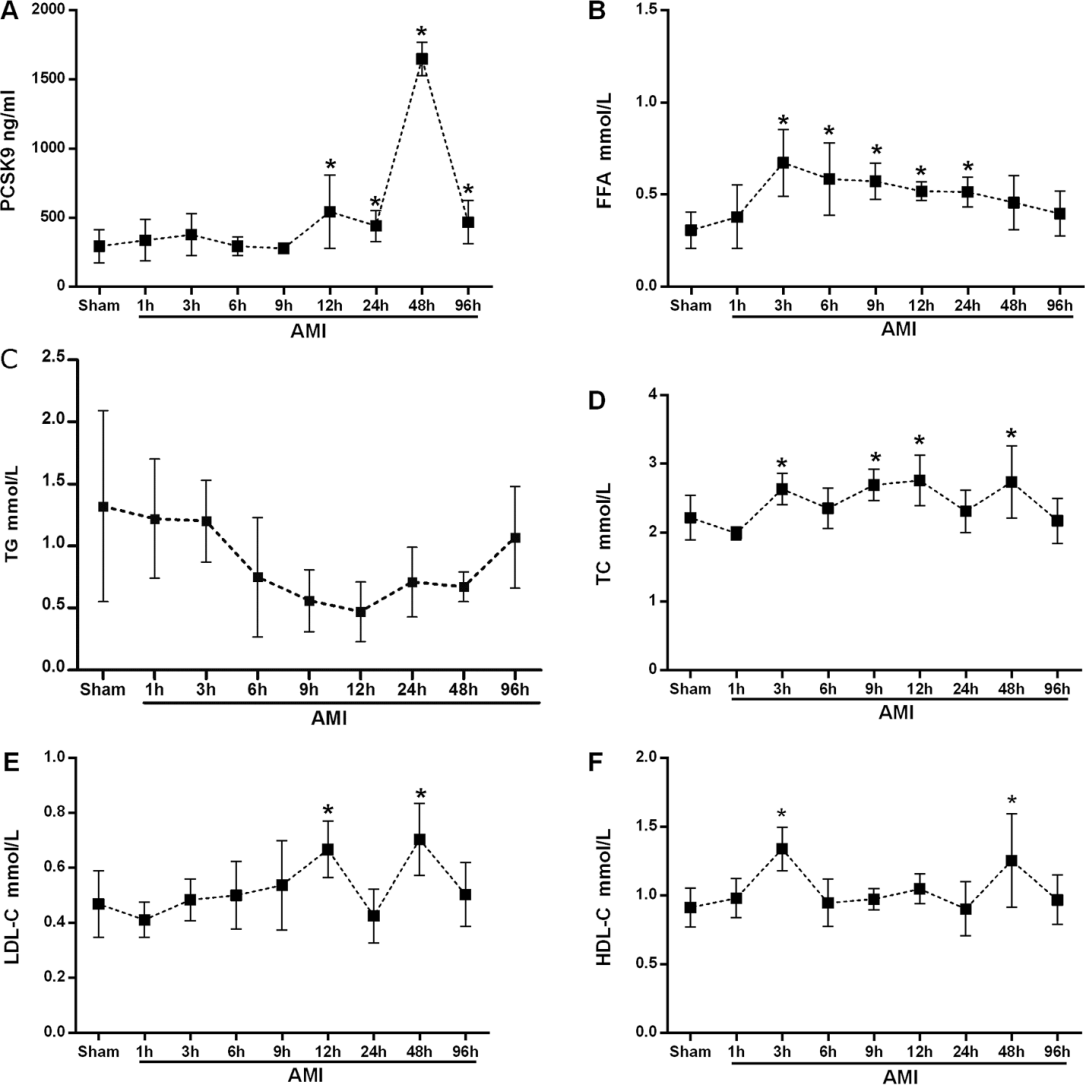
**Plasma levels of PCSK9 (A), FFA (B), TG (C), TC (D), LDL-C (E), and HDL-C (F) in sham and AMI groups.** *P < 0.05 vs. control. PCSK9 = proprotein convertase subtilisin/kexin type 9; FFA = free fatty acid; TG = triglyceride; TC = total cholesterol; LDL-C = low-density lipoprotein-cholesterol; HDL-C = high-density lipoprotein-cholesterol.

The plasma concentration of PCSK9 in sham and AMI groups were indicated in Figure 1. Compared with sham group, PCSK9 concentration was significantly increased at 12 h (544.18 ± 262.69 ng/ml vs. 293.53 ± 119.90 ng/ml, P < 0.05 vs. control) post AMI, and reached a peak level of 1647.29 ± 120.47 ng/ml (P < 0.05 vs. control) at 48 h, then decreased thereafter to reach a value of 467.54 ± 155.73 ng/ml (P < 0.05 vs. control) at 96 h. Meanwhile, we observed that the plasma WBCC level was increased at 3 h after infarction (6.32 × 10^9^/L vs. 4.24 ± 1.32 × 10^9^/L in sham group, P < 0.05 vs. control), and continued to rise at 6 h and 12 h (9.58 ± 2.26 × 10^9^/L, 12.74 ± 3.88 × 10^9^/L, respectively; both P < 0.05 vs. control).

### Plasma levels of lipid parameters

FFA level started to increase at 3 h after AMI, and maintained a high level until 24 h, then decreased to a level similar to that of the sham group. There was no significant change in TG level from 1 h to 96 h even though it showed a decrease and then regressive trend. The TC level exhibited an increase at 3 h after AMI, and thereafter maintained a high level with intermediate fluctuations at 6 h and 24 h. Its concentration returned to the sham level at 96 h. There was no significant change in LDL-C level from 1 h to 96 h except for two transient increase of LDL-C at 12 h and 48 h. HDL-C level was steady at most of the time point except for two transient increase at 3 h and 48 h.

### Expression of PCSK9 and LDLR mRNA in AMI rat hepatic cells

**Figure 2:**
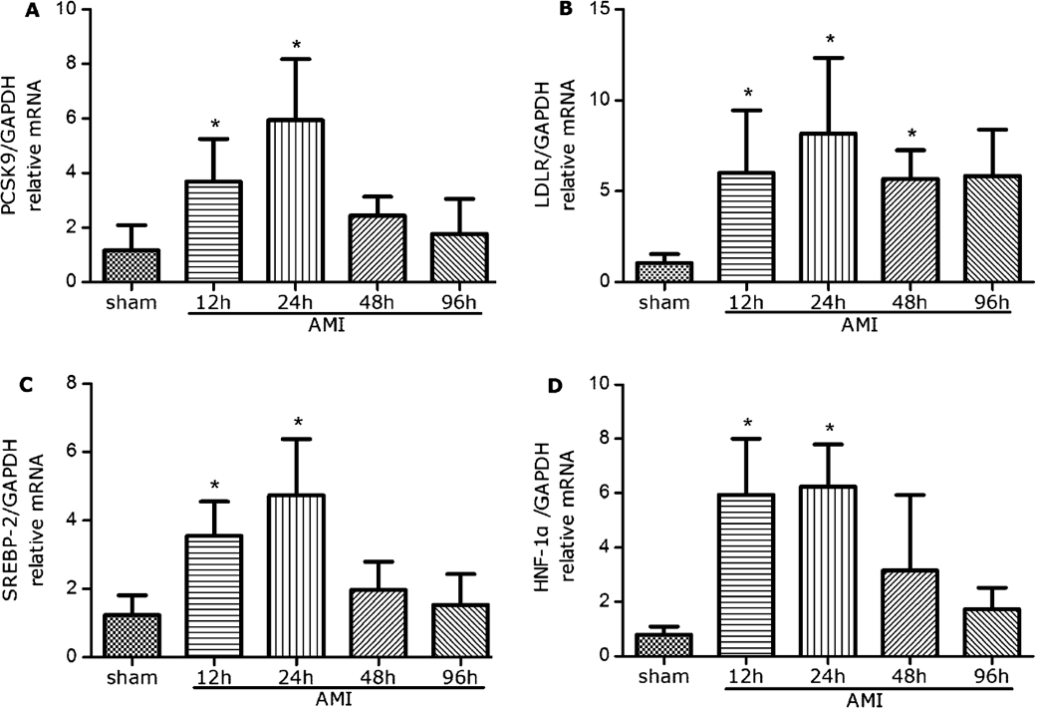
**The mRNA expression of PCSK9 (A), LDLR (B), SREBP-2 (C) and HNF1α (D) in the liver were measured using real-time PCR.** The relative ratio and standard deviation were calculated using comparative CT method (ΔΔCT value). All results were normalized against GAPDH. *P < 0.05 vs. control. PCSK9 = proprotein convertase subtilisin/kexin type 9; LDLR = low-density lipoprotein receptor; SREBP-2 = sterol regulator element–binding protein-2; HNF1α = hepatocyte nuclear factor 1α; GAPDH = glyceraldehyde 3-phosphate dehydrogenase.

To determine the relative mRNA expression of PCSK9 and LDLR in the liver at different time post AMI, RT-PCR was performed. As shown in Figure 2A-B, at 12 h and 24 h, the amount of PCSK9 mRNA was increased by 2.2-fold and 4.1-fold, respectively (P < 0.05 vs. control), then followed by a precipitous drop to a level similar to that of the sham group at 48 h and 96 h. Expression of the LDLR mRNA, which is known to be associated with PCSK9, was also increased by 4.7-fold at 12 h, and reached a peak level at 24 h (increased by 6.7-fold), but slightly decreased to 4.4-fold at 48 h (P < 0.05 vs. control). At 96 h, there existed no significant difference compared to the sham group.

### Expression of SREBP-2 and HNF1α mRNA in AMI rat hepatic cells

To determine the transcriptional level mechanism underlying the mRNA alteration of PCSK9 and LDLR at different period of AMI, we quantified SREBP-2 and HNF1α expression using RT-PCR. The mRNA expression of SREBP-2 (Figure 2C) and HNF1α (Figure 2D) were both up-regulated at 12 h (1.9-fold and 6.5-fold, respectively, P < 0.05 vs. control) and reached their peak level at 24 h (2.8-fold and 6.9-fold, respectively, P < 0.05 vs. control), then dropped to the level similar to the sham group since 48 h.

## Discussion

PCSK9 has been confirmed as a major factor regulating hepatic LDLR proteins and consequently serum cholesterol levels [7]. Recently, several studies have emphasized that PCSK9 was also involved in the physiology of non-hepatic cells or have LDLR-independent effects [15, 16]. Interestingly, in the present study our data for the first time found that the plasma PCSK9 concentration was drastically induced from 12 h to 96 h at the acute period of AMI in rat model. Paralleled with the enhanced plasma PCSK9 concentration, hepatic PCSK9 mRNA expression was also up-regulated by 2.2-fold at 12 h and 4.1-fold at 24 h. In consistent with previous studies, the up-regulated PCSK9 expression could be driven by SREBP-2 [21] and HNF1α [22], both of which are pivotal transcription factors for PCSK9.

The mechanisms accounting for the transiently up-regulated PCSK9 level at the acute period of AMI remain unclear. We speculated that it may be correlated with the following factors. Primarily, systemic inflammation may be one of the involving mechanisms. Inflammation is a hallmark throughout the distinct stages of atherosclerotic lesion formation preceding AMI as well as the time of plaque rupture [23]. Several large, prospective epidemiological, clinical and experimental studies converge on inflammation as a pivotal factor in AMI progression and exacerbation [24, 25]. In the study conducted by Yavuz MT et al. demonstrated that patients with significant coronary stenosis had elevated interleukin-6 (IL-6), high-sensitivity C-reactive protein (hs-CRP) and higher WBCC levels [26]. In the current study, we also observed that the plasma level of WBCC was dramatically increased at the early stage of AMI. Our previous clinical studies [27, 28] showed that a persistent and enhanced inflammatory responsiveness to pro-inflammatory stimulators, such as C-reactive protein, are involved in the pathogenesis of acute coronary syndrome. In a study neutrophil to lymphocyte ratio (NLR) was found to have a significant relationship with the severity of ACS assessed by SYNTAX Score and higher NLR was found in ST elevation myocardial infarction patients [29]. Activated neutrophils release a variety of proteolytic enzymes such as myeloperoxidase, which are responsible for tissue injury [30]. Therefore, it has been well established that the pathogenesis of AMI is accompanied with systemic inflammation. Additionally, the study conducted by Feingold KR et al. [16] demonstrated that in mice systemic inflammation induced by lipopolysaccharide or other treatments all resulted in a marked increase in hepatic PCSK9 mRNA levels (4 h-2.5 fold increase; 38 h-12.5 fold increase). Thus, it seems reasonable to consider inflammation as one of the underlying mechanisms. The second mechanism may be associated with the injury induced by AMI. Le Bras M et al. [17] revealed that plasma PCSK9 level was increased by 2-fold between days 0 and 8 in patients with severe multiple trauma, which may provide evidence that injury may be involved in the up-regulation of PCSK9 during the acute phase of AMI. Finally, necrosis and apoptosis [31] in the process of AMI may be the additional mechanism for the increased PCSK9 concentration. Similar to our study, Rousselet E et al. demonstrated that ischemic stroke triggered an up-regulation of PCSK9 level [32]. Additionally, Wu CY et al. using human endothelial cells demonstrated that oxidized LDL-induced apoptosis resulted elevated expression of PCSK9 and the apoptosis could be inhibited by PCSK9 siRNA [33]. It is plausible to consider that the above factors may contribute to the up-regulated PCSK9 level. However, detailed studies are warranted to explore the exact mechanisms involved in this specific pathological condition.

Although clinical implications of the elevated plasma PCSK9 concentration in the acute period of AMI are still vague, there may be several clinical significances. Firstly, PCSK9 may be an acute-phase response protein (APRP). In our study, we found that plasma PCSK9 concentration was remarkably induced from 12 h to 96 h post AMI. The impact of the increased PCSK9 concentration may be correlated to elevated serum LDL-C levels, which could have beneficial effects on host defense [34]. The changing pattern and relating impact of PCSK9 may be in accordance with standard APRP. However, Le Bras M et al. [17] showed that in patients with severe multiple trauma were lack of a rapid increase of circulating PCSK9 level, and posed a challenge to the hypothesis that PCSK9 is an APRP. Thereby, more investigations are required to verify this hypothesis. Secondly, PCSK9 may be a biomarker for future events of AMI. Previous study had demonstrated that at day 8, plasma PCSK9 concentrations were positively associated with clinical severity and acted as a late biomarker of severity illness in the severe trauma patients hospitalized in the intensive care unit [17]. Besides that, PCSK9 has been demonstrated to be expressed in human atherosclerotic plaques [35] and the study investigated the relationship between PCSK9 genotypes/haplotypes and severity of coronary atherosclerosis elucidated a modest association between minimum lumen diameter tertiles and haplotype 3 [18]. Based on these findings, we tentatively speculated that plasma PCSK9 concentration may be related to the infarct size and severity, which were predicting factors for cardiac remodeling [36] and even cardiac dysfunction. As a result, we suspected that PCSK9 may be a biomarker for the prognosis of AMI. Nevertheless, such hypothesis is being of under investigation in our group. Thirdly, the intensive statin therapy is an essential strategy after AMI [37]. Early administrated with statin can improve the outcome in those patients [38]. Considering the up-regulated PCSK9 expression by statin and AMI, adding PCSK9 inhibitors may bring greater benefits for those patients. Such hypothesis is also needed to be confirmed in the future.

Our study has several limitations. First, we did not observe the long-term impact of the enhanced PCSK9 expression on the prognosis parameters in the AMI rat model. Second, we did not evaluate the protein levels of PCSK9 and LDLR, which may provide more evidence about the relationship between PCSK9 and LDLR.

## Conclusions

In summary, we demonstrated for the first time that plasma PCSK9 concentration was significantly induced at 12 h after AMI, and still higher than the sham group at 96 h, and this result was further confirmed by the increased liver mRNA levels. At the transcriptional level, SREBP-2 and HNF1α were the predominate factors that activated the expression of PCSK9. The role of PCSK9 in myocardial injury may be needed further study.

## Abbreviations

PCSK9: Proprotein convertase subtilisin/kexin type 9
LDL-C: Low-density lipoprotein-cholesterol
LDLR: Low-density lipoprotein receptor
SREBP-2: Sterol regulator element–binding protein-2
AMI: Acute myocardial infarction.
